# Predictors of self-rated health: a 12-month prospective study of IT and media workers

**DOI:** 10.1186/1478-7954-4-8

**Published:** 2006-07-31

**Authors:** Dan Hasson, Bengt B Arnetz, Töres Theorell, Ulla Maria Anderberg

**Affiliations:** 1 Karolinska Institute, CRU/Dept of Neurobiology, Caring Science and Society, Karolinska University Hospital, Eugeniahemmet, T4:02, 171 76 Stockholm, Sweden; 2 Uppsala University, Department of Public Health and Caring Sciences, Section for Social Medicine/CEOS, Uppsala Science Park, SE-751 85 Uppsala, Sweden; 3 IPM – The National Swedish Institute for Psychosocial Medicine, Granits väg 8, SE-171 77 Stockholm, Sweden; 4 Division of Occupational and Environmental Medicine, Wayne State University, Detroit, Michigan, 48201-2011, USA

## Abstract

**Objective:**

The aim of the present study was to determine health-related risk and salutogenic factors and to use these to construct prediction models for future self-rated health (SRH), i.e. find possible characteristics predicting individuals improving or worsening in SRH over time (0–12 months).

**Methods:**

A prospective study was conducted with measurements (physiological markers and self-ratings) at 0, 6 and 12 months, involving 303 employees (187 men and 116 women, age 23–64) from four information technology and two media companies.

**Results:**

There were a multitude of statistically significant cross-sectional correlations (Spearman's Rho) between SRH and other self-ratings as well as physiological markers. Predictors of future SRH were baseline ratings of SRH, self-esteem and social support (logistic regression), and SRH, sleep quality and sense of coherence (linear regression).

**Conclusion:**

The results of the present study indicate that baseline SRH and other self-ratings are predictive of future SRH. It is cautiously implied that SRH, self-esteem, social support, sleep quality and sense of coherence might be predictors of future SRH and therefore possibly also of various future health outcomes.

## Background

Self-rated health (SRH) is one of the most widely used single measures of perceived current health status [1-9]. In spite of variation in wording of the question, there is extensive evidence that SRH is a potent predictor of future survival/mortality and morbidity [5,10], functional decline and disability and utilization of health care [1,3,10]. Most previous studies have shown that SRH is an independent predictor of future health outcomes, even after adjusting for self-ratings of other health-related measures, physician-reported health status, behavioural and psychosocial risk factors, socioeconomic status and environmental factors. Nevertheless, debate still continues about what SRH really represents [1,2,4,5,7,10,11].

It has been proposed that SRH represents an individual's general perception of health, including biological, psychological and social dimensions. Therefore SRH might be more sensitive in health monitoring than external measures of health [9]. Furthermore, it has been indicated that risk associated with poor SRH status is higher than that associated with poor objective health measures [12]. Kaplan & Camacho [6] on the other hand, have found that objective health status has a stronger relationship with mortality than SRH.

In a previous article, we reported about some short-term (six months) beneficial effects of a web-based stress management and health promotion system on psychological and physiological indicators of health, stress and recovery [13]. There is, however, a lack of knowledge about whether or not there are specific population groups that benefit more respectively less from web-based interventions, with regard to SRH. In addition, more knowledge is needed as to which factors predict long-term variation or trends in SRH, and if the web-based health promotion intervention can predict future SRH.

Most studies on SRH are of cross-sectional design. The findings of significant associations between different variables are more difficult to interpret compared to longitudinal studies with repeated measures. Consequently, it has been repeatedly shown that SRH correlates with a multitude of biological, psychological and social factors. Only few studies have examined predictors of future SRH longitudinally and combined the measure with other subjective and/or objective variables [1,4,8-11,14]. Still fewer have combined assessments of sociodemographic, psychosocial, and behavioural determinants at the same time [9,10,14]. Therefore, there is a need to comprehensively and prospectively monitor predictors of future SRH. Most commonly, SRH has been used as a predictor for different kinds of dependent variables or outcomes. It has been reported that SRH possesses considerable predictive validity [1]. More knowledge is needed however, as to possible predictors of SRH itself over time.

Therefore, the aim of the present study was to determine health-related risk and salutogenic factors and to use these to construct prediction models for future SRH, i.e. find possible characteristics predicting individuals improving or worsening in SRH over time.

## Methods

### Participants

In collaboration with a White-Collar Union (Sif) and a Swedish Employers' Association (Almega), ten companies insured by the study's source of funding Alecta (an occupational pension plan company) were asked as to their interest in participating. These companies were selected and contacted by employees at Alecta, by mail and phone. The management departments of six out of the ten companies were interested. Informed of the basic inclusion criteria, i.e. minimum group of ten individuals and access to economic production data, 2–4 departments within each company were chosen and asked by the company management as to their interest in participating. The managers of the chosen departments in turn asked their employees whether they were interested in participating. No incentives were offered to the participants, with exception of the extensive blood sampling including feedback, which seemed to be a motivator for many participants.

There is no information on the exact number of employees that were asked to participate in the study. An exception was one of the media companies where 95 out of 100 possible participants chose to participate. In general there was also a great interest from the other departments and similar participation rates is therefore estimated. Altogether, 317 participants from 22 departments/units in four information technology and two media companies enrolled in the study. The participating departments were, within each company, randomized by lottery to either the intervention or reference group. Fourteen participants were excluded because of communication-related problems (n = 7), change of mind in willingness to participate or leaving job before initiation of the study (n = 7). Thus, 303 persons finally participated in the study, out of which 26 participants (8.6%) dropped out. The reasons for dropping out were job termination (n = 7), change of workplace (n = 2), foreign service/moving abroad (n = 6) or other reasons (n = 11). There were no significant differences in dropout rates between the groups (6.9% in the intervention group vs. 9.8% in the reference group, p between groups = n.s.). There were no significant differences between the intervention and reference groups in socioeconomic background or psychophysiological measures at baseline. The participants had professions such as IT technicians, programmers, system developers as well as journalists/reporters, news presenters, sound technicians and photographers. The main type of work-site was open plan offices. Many participants from the IT-companies were partly located in the work sites of their customers for longer or shorter periods. For the media companies, some participants, such as photographers and reporters, were partially ambulatory and worked in different locations. The common feature for all participants was regular and daily computer usage at work.

Out of the 303 participants, only the participants that had complete SRH scores from the baseline and 12-month follow-up (n = 230) measurement were selected for the final analyses. Consequently, participants with missing values in the first or last measurement were not included in the regression analyses and ANCOVA. More detailed information about the measurements is presented below.

### The web-based tool

Table 1 provides a detailed description of the web-based tool and illustrates similarities and differences in the features that were offered to the intervention and reference group respectively. A web-based tool for health promotion and stress management was developed and offered all participants real-time monitoring of perceived current health and stress status, a diary and information about stress and health (Table 1). In addition, participants in the intervention group were offered web-based cognitive exercises, aimed at decreasing unwanted stress and promoting health and recovery through health promotion initiatives. The exercises included techniques for relaxation, time management, cognitive reframing and a chat. Thus, the only things that distinguished the groups were the addition of the cognitive exercises and the chat in the intervention group. The web-based tool was developed by the researchers and most techniques are commonly utilized techniques in cognitive and behavioral psychology and stress management. These techniques were modified so that they could become more or less self-instructing to be used for self-help purposes. Exposure to the intervention for both groups could only be logged via the number of logins to the website.

**Table 1:** Features of the web-based tool for the study groups respectively.

**Feature**	**Intervention group**	**Reference group**
*Monitoring tool for stress and health levels with instant feedback; graphs illustrating current and retrospective ratings and an option to compare results with other groups with the same socioeconomic profile, within the same department/company and all the respondents in the data base. The questionnaire was compiled by a ten-item questionnaire for regular or daily usage*.	YES	YES
*Diary connected to the monitoring tool so that ratings and notes could be compared and examined retrospectively. The diary could be used as stress management but also as a tool for improving self-knowledge and how different events affect health and well-being*.	YES	YES
*Popular scientific information on stress and health compiled by various Swedish researchers*.	YES	YES
*Self-help in the form of classical stress management exercises for; relaxation and sleep improvement, cognitive reframing, time-management, emotional control and self-knowledge, strengthening self-esteem, life reflection, dissociation*.	YES	**NO**
*Chat*	YES	**NO**

### Questionnaire

A questionnaire was compiled and included about 100 questions concerning socioeconomic status, consumption of caffeine drinks, expectations about the research project, self-rated health (SRH), stress and wellbeing at work as well as during leisure time, health economics and performance at work (Table 2). Most of the questions were presented as Visual Analogue Scales (VAS) and some, concerning health economy, work time, basic daily functioning and symptoms of ill health, were presented as multiple-choice questions. Most of the newly constructed single VAS questions were based on previously validated Likert-based items or indices [15-20]. Participants filled out the questionnaire online at baseline (before the initiation of the study), at the end of the six-month intervention and at a long-term follow-up 12 months after baseline.

**Table 2:** Theoretical models, items and topics covered by the questionnaire.

**Models**	**Topics – generalized self-ratings.**
*Socioeconomic and background factors*	Age, sex, annual income and self-rated financial situation, educational level, marital status, possession of children, work role (co-worker, middle-manager, manager), amount of customer contact, duration of current working position, smoking habits, satisfaction with eating habits, consumption of coffee, tea, soft drinks and energy drinks. Expectations of the possible effects of the research project on stress and health level.
*Lifestyle, health promoting and compromising behaviours, cognitive function, sense- of-coherence and wellbeing*.	Self-rated health (last year, right now and future expectations), sleep quality, memory, concentration ability, ache in various body parts, physical exercise habits, mental energy, frequency and source (home, work or combination) of stress, stress management ability, satisfaction with leisure-time, life goals, communication ability with others, meaningful life, future optimism/pessimism, flexibility, daily computer, phone and cellular phone usage, social support, reflection on health improvement.
*Work-related factors, demand/control, effort/reward*,	Work satisfaction, efficiency, competence (sufficiency, development, usage), meaningful work, work atmosphere, work intensity, number of breaks during a regular working day, average working hours and distribution over the week (actual and desired), flexibility of work, general mood on the way to work (sad – happy), working effort, work reward, influence on work situation, work stress, work confidence, support from managers, collegial support, work-place goal clarity and realism, work-place efficiency, reflection on efficiency improvement, priority between health and achievement, time perspectives on decisions at work, existence of serious considerations to quit job, number of sick-leave days, health-economic aspects.

Most items were presented as "straight forward" VAS, e.g. How is your overall sleep quality (Very poor – Very good). For complete questionnaire please e-mail your request to: dan.hasson@ki.se

### Blood sampling

The complete list of biological markers analyzed in the current study is presented in Table 3. More biological markers of general nature, such as blood status, were collected for overall health matters or all-purpose profiling. However, these markers were not analyzed in the current study. The biological markers analyzed in the present study were the ones that could be related to various stress- or health-related hypotheses.

**Table 3:** Complete list of physiological markers collected at baseline and 6 and 12-month follow-up.

**Categories**	**Physiological marker**
*Cardiovascular system and lifestyle*	Blood pressure, pulse, waist-hip ratio, BMI, P-BNP (brain natriuretic peptide), P-PAI-1 (plasminogen activator inhibitor 1), insulin, B-HbA1C, S-triglycerides, S-cholesterol, S-HDL, S-LDL, P-fibrinogen, B-trombocytes.
*Stress-related (HPA-axis, catabolic)*	S-prolactin, P-ACTH (adreno corticotropic hormone), S-Cortisol, S-TSH (thyroid stimulating hormone), S-T3, S-T4 (free), S-urate.
*Recovery-related (anabolic)*	S-growth hormone, S-IGF-1, S-DHEAS-S (dehydroepiandosterone sulphate), S-estradiol, S-testosterone, S-SHBG (sexual hormone binding globulin).
*Immune markers and neuropeptides*	S-IL-1beta, S-TNFα (tumour necrosis factor alpha), S-CRP, high sensitive (c-reactive protein), P-substance P, P-NPY, CgA (P-chromogranin A), B-LPK (leukocyte particle concentration).

Blood samples were collected from study participants between 7.00–11.30 am at each specific worksite (or nearby). Questionnaires were filled out during the same time period (usually same day or week) in order for the outcome of the blood and questionnaire data to be as comparable as possible. The exact time for blood sampling were recorded for each participant at baseline and at the end of the study so that the blood could be collected at the same time (± 15 minutes). Participants were instructed not to eat or drink (except water), nor use nicotinic substances at least ten hours before blood sampling.

### Self-rated health measurement

For the present study the question: "How is your health at the moment?" was used and the VAS anchors were "very poor" on the left end and "very good" on the right end. It has been proposed that repeated measurements of variables, such as SRH, could benefit by being assessed using a VAS, since the VAS is probably more responsive, i.e. sensitive to detect clinically significant change (as distinct from statistically significant), compared to a four or five point Likert scale. Furthermore, it has been suggested that VAS have several other advantages compared to the Likert scale, especially with regard to repeated measures [21-29]. For example, the VAS compared to the Likert scale, seems to exhibit less end-aversion bias, for some groups be easier to use and understand. In any case, we have previously compared scorings on SRH for respondents using VAS as well as the more traditional Likert based item and found that the items were more or less comparable and interchangeable [22].

### Statistical analyses and validity

The program SPSS 13.0 for windows was used for statistical analyses. Initially, all variables were assessed for normality using Kolmogorov-Smirnov test. Thereafter, a new variable was created to assess possible differences in baseline means/ranks between participants maintaining/improving (SRH_12 months _≥ SRH_baseline_) or worsening (SRH_12 months _< SRH_baseline_) in SRH over time. The parametric Unpaired samples t-test and the non-parametric Mann-Whitney U test were utilized for this purpose. SRH at the 12-month follow-up served as the dependent variable in the regression analyses. For the non-parametric logistic regression, the variable was divided by quartile split into high (top quartile) and low (remaining quartiles) categories.

In a previous article, we reported that SRH had increased in the whole study group (intervention vs. reference group) with no statistically significant difference between the groups at the post-intervention follow-up, i.e. a six months from baseline [13]. In that study, as well as in this, two-way analysis of covariance (ANCOVA) was used to illustrate changes over time (time, group and group × time). ANCOVA adjusts for initial differences so that the results more precisely reflect the difference in trends between the groups, and thus permits a more sensitive analysis compared to regular analysis of variance (ANOVA). The increase in sensitivity arises from the fact that the covariance reduces the error term (within-group variability) against which trends in SRH are compared. Furthermore, ANCOVA is not very sensitive to small deviations from a normal distribution [30]. In the present study, baseline value of SRH was used as covariate.

Criterion validity refers to the degree to which a variable converges (convergent validity) or discriminates (discriminant validity) between measures that should be related and non-related respectively. Spearman's rank correlations test was used for this purpose in a cross-sectional analysis of the baseline measurement. It was expected that the findings of the present study would be congruent with previous studies with cross-sectional design, where SRH has been related to socioeconomic factors as well as biological, environmental and psychosocial factors.

Logistic and linear regression analyses were used to model the probability of maintenance/improvement and worsening in SRH over time. The regression models were estimated in four steps to address the questions if clinical/physiological measures/risk factors predict future SRH and if these variables retain their importance after adjustment for other self-reported measures. For the logistic regression, all variables were divided by quartile split into high and low categories to render comparable odds ratios. The top quartile was labelled "high" and the remaining quartiles "low". The independent variables, i.e. questionnaire items and physiological markers, were selected with the primary rationale to choose variables that previously have been associated with SRH. The secondary rationale was to choose isolated variables that were significantly associated with SRH in the cross-sectional analysis. In Table 4, the variables entered at each step in the regression analyses are presented.

**Table 4:** Independent variables entered in the different steps of the logistic and linear regression analyses.

**Step (methods logistic/linear regression)**	**Independent variables at baseline**
Step 1 (Enter)	**Socioeconomic variables: **Age, sex, education, annual income, study group (intervention vs. reference group).
Step 2 (Forward conditional/Forward)	**Behavioural factors: **Number of logins to the website, smoking habits, alcohol consumption, eating habits, physical activity, working hours.
Step 3 (Forward conditional/Forward)	**Psychosocial/cognitive/personality factors**: SRH, sleep, concentration ability, mental energy, stress frequency, stress management ability, leisure time, goals, sense of coherence, belief in the future, self-esteem, social support, work mood, reward, and ability to influence work.
Step 4 (Forward conditional/Forward)	**Physiological markers: **BMI, triglycerides, cholesterol, HDL, CRP (c-reactive protein), DHEA (dehydroepiandosterone), GH (growth hormone), cortisol, TSH (thyroid stimulating hormone), fibrinogen.

Dependent variables were SRH_increase vs. worsening _& ΔSRH_12 months-baseline _

Factors such as socioeconomic status, marital status and gender are known to be associated with outcomes in health, and were therefore included as covariates in the first step of the regression analyses. Also group (intervention vs. reference) was included as a factor in the first step to adjust for possible study group effects. For the subsequent steps in the regression analyses, VAS-items from the questionnaire and physiological markers were used as independents. The rationale for the second step was to adjust for factors that might disturb the relationships, i.e. health-related behavioural factors. In the third step, factors important for mental and physical health and wellbeing were included. The fourth and final step included physiological variables.

### Role of the funding source and ethical approval

The funding source had no involvement in the study design; in the collection, analysis, and interpretation of data; in the writing of the report; and in the decision to submit the paper for publication. The ethics committees of Uppsala University (Dnr 01–188) and Karolinska Institute (Dnr 01355) approved the research project.

## Results

Altogether, 230 participants were rendered for the final analyses as they had complete SRH scorings from the baseline and 12-month follow-up measurement. 73 participants had missing values in one or both measurement occasions, and were thus precluded from further analyses. The means in SRH for the total sample at baseline was 66.5 (SD 21.4; n = 260), after six months 68.5 (SD 20.9; n = 258) and after twelve months 68.6 (SD 21.8; n = 253). Paired samples t-tests between the baseline and 12-month follow-up measurement revealed that these differences were not statistically significant. Table 5 depicts the baseline SRH means and medians divided by the main background and socioeconomic factors.

**Table 5:** Baseline SRH means and medians divided by the main background and socioeconomic factors (N = 260).

**Background factor**	**SRH Mean (SD)**	SRH Median (IQR*)	**N**
**Age**			
≤ 30 yrs	64 (± 22)	69 (47–78)	68
31–45 yrs	67 (± 20)	69 (51–s83)	96
≥ 46 yrs	68 (± 23)	71 (54–87)	96
**Sex**			
Male	66 (± 21)	69 (50–82)	157
Female	67 (± 23)	73 (54–86)	103
**Edu cation**			
Compulsory/high school	65 (± 20)	68 (50–82)	128
Academic degree	68 (± 22)	72 (58–85)	132
**Annual income**			
< 25,000 USD	60 (± 23)	64 (47–78)	56
25,000 – 40,000 USD	68 (± 21)	72 (53–84)	157
> 40,000 USD	70 (± 19)	71 (60–87)	47
**Marital status**			
Married/co- inhabiting/liveapart	68 (± 20)	71 (56–83)	206
Single	60 (± 25)	63 (45–82)	54

*IQR = Inter Quartile Range

The parametric Unpaired samples t-test and the non-parametric Mann-Whitney U test revealed that there was a statistically significant difference in baseline ratings of SRH between the participants maintaining/improving and those worsening in SRH. Participants worsening in SRH had a higher (better) baseline mean or rank compared to those maintaining/improving (75.4, SD 17.6 vs. 60.4, SD 21.4; p < .0001).

**Figure 1:**
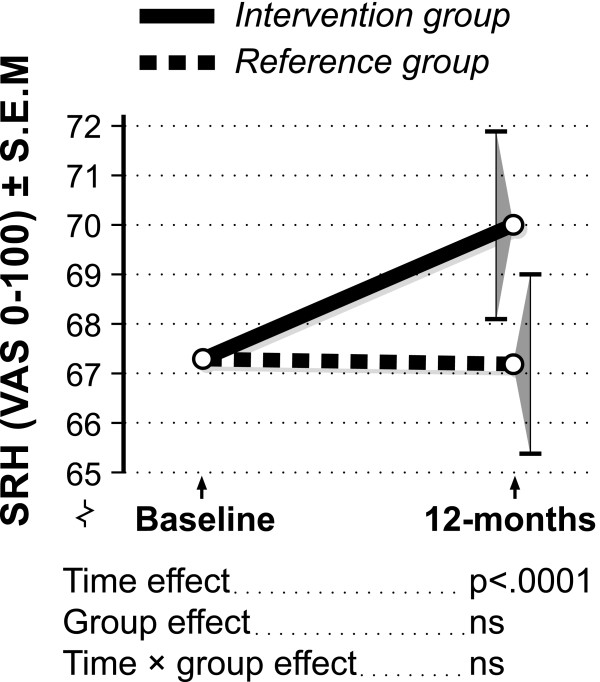
Two-way ANCOVA. Changes in SRH between baseline and the 12-month follow-up.

Figure 1 depicts the results from the Two-way ANCOVA, i.e. change in SRH for the intervention and reference group respectively. There was a statistically significant improvement for the group as a whole between the first and last measurement and no statistically significant difference between the intervention and reference group (time effect p < .0001, time × group effect NS). Thus, both groups improved ratings of SRH over time.

### Validity

Criterion validity was assessed using Spearman's Rank correlation test. Baseline SRH significantly correlated with numerous variables that are known to be related, indicating good overall criterion validity (Table 6).

**Table 6:** Significant correlations between baseline SRH and other baseline measures.

**Significantly related baseline measures (VAS)**	**Self-rated health at the moment**		
	Correlation coefficient†	p (2-tailed)	*n*
Self-rated financial situation.	.263**	.000	260
Annual income.	.144*	.020	260
Satisfaction with eating habits.	.359**	.000	260
Self-rated health last year.	.765**	.000	260
Expected health in one year.	.704**	.000	260
Value about having complete health proportionally to other things in life.	.237**	.000	260
Sleep quality.	.289**	.000	260
Memory.	.294**	.000	260
Concentration ability.	.416**	.000	260
Physical activity.	.138*	.026	260
Mental energy.	.275**	.000	260
Frequency of stress.	-.209**	.001	260
Stress management.	.271**	.000	260
General satisfaction with leisure time.	.275**	.000	260
Existence of goals in life.	.172**	.006	260
Self-rated communicational skills/ability.	.230**	.000	260
Sense of coherence.	.340**	.000	260
Optimism towards future.	.366**	.000	260
Self-confidence/self-esteem in general.	.302**	.000	260
Social support.	.255**	.000	260
Overall experience of work.	.179**	.004	260
Work efficiency in general.	.200**	.001	260
If one's competence is utilized at work.	.213**	.001	260
Feel that one's work is meaningful.	.154*	.013	260
Work atmosphere.	.144*	.020	260
Work flexibility.	.256**	.000	260
Emotion on the way to work? (sad – happy)	.215**	.000	260
If one is properly rewarded for their working efforts.	.124*	.046	260
Ability to influence work situation.	.197**	.001	260
Confidence at work.	.172**	.005	260
Social support from work colleagues.	.189**	.002	260
Clear goals at work.	.155*	.013	256
Realistic goals at work.	.217**	.000	256
Work place efficiency.	.254**	.000	260
Sick days during the last year.	-.294**	.000	260
Amount of consideration about one's health.	.150*	.015	260
Pains/inconveniences.	-.322**	.000	260
Fears/melancholy.	-.345**	.000	260
BMI (body mass index).	-.138*	.035	233
Waist-Hip ratio.	-.159*	.016	230
S-CRP (c-reactive protein) high sensitive.	-.124*	.048	256
S-SHBG (sexual hormone binding globulin).	.146*	.020	256
S-T4 (thyroxine) free.	.178**	.004	256
P-Fibrinogen.	-.155*	.013	256
B-LPK (leukocyte particle concentration).	-.133*	.033	256

** Correlation is significant at the 0.01 level 2-tailed.

* Correlation is significant at the 0.05 level 2-tailed.

### Regression analyses

Table 7a–b presents the results from the logistic and linear regression analyses. In the logistic as well as linear regression baseline levels of SRH was a major predictor of future SRH (OR = 4.398, 95% CI 1.834–10.543, p = .001). Hence, participants in the top SRH quartile at baseline were approximately 4.4 times more likely remain in the top quartile at the 12-month follow-up. Furthermore, in the logistic but not in the linear regression, self-esteem (OR = 2.679, 95% CI 1.018–7.051, p = .046) and social support (OR = 2.572, 95% CI 1.001–6.609, p = .05) significantly predicted future SRH. Thus participants with highest baseline ratings (top quartile) of self-esteem and social support were approximately 2.6 times more likely to exhibit maintenance or improvement in SRH at the 12-month follow-up. Finally, the logistic regression revealed two trends that failed to reach statistical significance at the .05 level when adjusting for other factors in the final model. However, these variables were statistically significant predictors in a previous step of the analysis. The trends indicate that a higher alcohol intake at baseline increases the probability of belonging to the lower SRH quartiles at the 12-month follow-up with approximately 44%. Moreover, individuals with the highest baseline ratings of physical exercise were approximately 2.5 times more likely to remain in the top quartile at the 12-month follow-up. The logistic regression model correctly predicted 82.6% of future SRH (93.8% in the low SRH quartile and 44.7% in the top quartile).

**Table 7a:** Final logistic regression model predicting SRH at the 12-month  follow-up (n = 230).

**Predictors**	**OR**	**95,0% CI for OR**	**p-value**
Age	.825	.303 – 2.251	ns
Sex	1.547	.595 – 4.022	ns
Educational level	1.255	.487 – 3.234	ns
Annual income	2.812	.929 – 8.508	.067
Group (intervention vs. reference)	1.409	.570 – .3.482	ns
Alcohol consumption*	.557	.176 – 1.757	ns
Physical exercise*	2.479	.895 – 6.872	.081
Baseline SRH	4.398	1.834 – 10.543	.001
Self-esteem	2.679	1.018 – 7.051	.046
Social support	2.572	1.001 – 6.609	.05

Nagelkerke r^2 ^was 36% for the final logistic regression model.

* These variables are part of the final regression model in spite of failure to reach statistical significance. This is due to the fact that they were significant in a previous step of the analysis and have become statistically insignificant when adjusting for other incoming variables.

**Table 7b:** Final linear regression model predicting SRH at the 12-month  follow-up (n = 230).

**Predictors**	**Adjusted r**^2 ^**%**	**Standardized Beta**	**95% CI for Standardized beta**	**F**	**p-value**
*The whole model*	*27.4*			*10.809*	*<.0001*
Age	*	.096	-.885 – 6.363		ns
Sex	*	-.019	-6.199 – 4.522		ns
Educational level	*	.084	-1.532 – 8.883		ns
Annual income	*	-.044	-6.188 – 3.023		ns
Group (intervention vs. reference)	*	-.074	-8.453 – 1.980		ns
Baseline SRH	20.3	.342	.228 – .507		<.0001
Baseline sleep quality	5.5	.233	.092 – .310		<.0001
Baseline sense of coherence	1.6	.153	.026 – .291		.02

* The aggregated adjusted r^2 ^for these variables is about 1%.

In addition to lower baseline ratings of SRH, the linear regression identified higher baseline ratings of sleep quality and sense of coherence as significant predictors of higher SRH at the 12-month follow-up. Altogether, this model accounted for 27.4% of the explained variance. Socioeconomic variables were not significant predictors in any of the regression models.

## Discussion

The aim of the present study was to determine predictors of future SRH. The study period was one year and conducted on a working population from IT and media companies. The logistic regression identified baseline scorings of SRH, self-esteem and social support to be significant predictors of future SRH. The linear regression also revealed baseline ratings of SRH, sleep quality and sense of coherence as significant predictors of future SRH.

### Validity

The cross-sectional correlation analysis revealed that SRH significantly correlated with other self-rated and objective measures. Most of these associations have been confirmed in previous studies [1-3,5,10,14]. This finding indicates a good overall criterion validity of the data in the present study. However, differences in methodology, settings and statistical analysis strategies make it difficult to reliably compare the results of different studies on SRH [4]. Thus, it is concluded that the level of validity of the present study at least should be similar with that of previous studies.

An interesting finding was that there were some moderate to strong correlations between baseline SRH and other baseline measures, such as leisure time, social support, stress management, mental energy and self esteem (positive) and between SRH and frequency of stress, pain, BMI and fears/melancholy (negative). These findings have all been confirmed in the different studies cited in the above paragraph. It was also interesting to note that there were negative correlations between SRH and biological markers such as CRP and fibrinogen. This finding confirms what we have found in clinical practice, where increased levels of CRP and fibrinogen are common in patients with stress-related syndromes.

### Regression analyses

One might expect that some of the socioeconomic variables would have predicted future SRH. In this sample, however, the contrasts between the participants are undoubtedly smaller than in the normal population, rendering a lesser variance. This might explain why the socioeconomic variables were not significant predictors of future SRH in this study. However, annual income was a marginally significant predictor in the final logistic regression model. Furthermore, one year is a rather short time to expect significant predictive effects from these otherwise predictive variables.

With exception for SRH, the logistic and linear regression analyses yielded different predictors. One possible partial explanation might be that the dichotomization of variables needed for logistic regression decreases the variance and statistical power compared to the linear regression that retains more of the variance. Moreover, the chosen cutoff is not necessarily the biologically correct one. Another explanation is that SRH accounts for a major part of the variance. In fact, when the baseline SRH variable was removed as a predictor, the regression analyses showed more similar results. Significant predictors in the logistic regression were then physical activity, sense of coherence and social support, and in the linear regression, sleep quality, sense of coherence and social support.

Self-esteem and social support as well as sleep quality and sense of coherence has previously been identified both as predictors and/or mechanisms of maintaining and improving health and general wellbeing [31-39]. These factors are important functions for successful stress management, and it has been implied that psychosocial states, including stress, are strongly related to SRH [1,2,40].

The strongest predictor of future SRH was baseline SRH. Findings from previous prospective studies confirm the result of this study, which implies that the results of this study may be valid [9,10]. For example, also Bailis and colleagues (2003) found that SRH was, independently of other measures, the strongest predictor for SRH two years later. The predictive strength of SRH on future SRH and other health outcomes may indicate at least a short term stability of the measure itself and perhaps a low overlap with other possible predictors. Furthermore, as both this and most previous studies imply, clinical factors are less powerful predictors of SRH compared to other kinds of self-ratings.

Finally, it is particularly important to address one of the findings of the present study. SRH improved in both study groups (intervention vs. reference; time effect but no significant time × group effect) at the 6 and 12 months post-intervention follow-ups respectively. However, as shown in the regression analyses in the present study, "study group" was not a significant predictor in any of the regression models. This implies that the web-based intervention neither had short-term nor long-term effects on SRH. Thus, the result may indicate that primarily SRH, but also sleep quality and sense of coherence are more profound indicators of health status, and that SRH is not easily affected by temporary circumstances. Rather, it might be affected on a longer-term basis by general health status, total load of life, life-events or other burdens that have not been captured by the regression analyses or measures assessed in the present study. For example, one partial explanation might be that the active intervention period, i.e. 0–6 months, was conducted during a turbulent time. This general turbulence was soothed during the 12-month follow-up, which partly and generally could explain an improvement in SRH. Furthermore, sleep quality was one of the factors that improved in the intervention group compared to the reference group at the 6-month follow-up. Perhaps participants that for different reasons were able to sustain a good sleep benefited more with regard to SRH. However, as for now, it is not possible to draw other reliable conclusions than that the intervention had no long-term effect on SRH.

### Methodological considerations

There are some methodological considerations that have to be addressed. With regard to generalizability of the results it is important to note that the study group consisted of a healthy, working sample from IT- and media companies. Therefore the results of this study may not be generalizable to for example a sample of individuals suffering from different long-term disorders and diseases or an elderly, retired sample.

### Future implications

A multitude of possible predictors of SRH have been found in previous studies. This study confirms some of the previous findings. Future research should preferably focus on finding explanations and consistent patterns of predictors of future SRH. An interesting aspect that could render valuable information is to assess predictors of individuals that fluctuate in SRH over time. It would be interesting to know if fluctuation per se in SRH is a salutogenic or pathogenic pattern on a long-term basis. There is also a need to develop and assess psychophysiologically based explanatory models about the predictive ability of SRH on future health outcomes.

## Conclusion

The results of the present study and others indicate that SRH and other self-ratings are predictive of future subjective and objective health outcomes. However, different settings, types of population and methodology and statistical analyses strategies of various studies examining SRH makes it difficult to compare results and therefore to draw reliable conclusions. This study cautiously implies that SRH, self-esteem, social support, sleep quality and sense of coherence might be predictors of future SRH and therefore possibly also of various future health outcomes.

## Competing interests

Following the termination of this study, BA and DH have commercialized the web-based health promotion and stress management tool. The titles referring to any of the persons/authors involved in this article are made without any considerations to ownership to possible intellectual property rights.

## Authors' contributions

BA was the principal project designer as well as the project leader and DH accomplished the practical study logistics. DH conducted the statistical analyses and drafted the manuscript, with substantial and essential input from TT, UMA and BA. All the authors have read and approved the final version of the manuscript.
